# Fertility Preservation in Cancer Patients: *In Vivo* and *In Vitro* Options

**DOI:** 10.22074/cellj.2016.4880

**Published:** 2017-02-22

**Authors:** Rouhollah Fathi, Mojtaba Rezazadeh Valojerdi, Bita Ebrahimi, Farideh Eivazkhani, Mahzad Akbarpour, Leila Sadat Tahaei, Naeimeh Sadat Abtahi

**Affiliations:** 1 Department of Embryology, Reproductive Biomedicine Research Center, Royan Institute for Reproductive Biomedicine, ACECR, Tehran, Iran; 2 Department of Anatomy, School of Medical Sciences, Tarbiat Modares University, Tehran, Iran; 3 Department of Pediatrics, Pritzker School of Medicine, University of Chicago, Chicago, USA

**Keywords:** Fertility Preservation, Cryopreservation, Cancer, Transplantation, Ovarian Follicle Culture

## Abstract

Oocyte, embryo and ovarian tissue cryopreservation are being increasingly proposed for fertility preservation among cancer patients undergoing therapy to enable them to have babies after the cancer is cured. Embryo cryopreservation is not appropriate for single girls without any sperm partner and also because oocyte retrieval is an extended procedure, it is impossible in cases requiring immediate cancer cure. Thus ovarian tissue cryopreservation has been suggested for fertility preservation especial in cancer patients. The main goal of ovarian cryopreservation is re-implanting the tissue into the body to restore fertility and the hormonal cycle. Different cryopreservation protocols have been examined and established for vitrification of biological samples. We have used Cryopin to plunge ovarian tissue into the liquid nitrogen and promising results have been observed. Ovarian tissue re-implantation after cancer cure has one problem- the possibility of recurrence of malignancy in the reimplanted tissue is high. Xenografting-implantation of the preserved tissue in another species- also has its drawbacks such as molecular signaling from the recipient. *In vitro* follicle culturing is a safer method to obtain mature oocytes for fertilization and the various studies that have been carried out in this area are reviewed in this paper.

## Introduction

Every year a large number of people worldwide is diagnosed with different types of cancer. 

Many of them are women and young girls who are within the reproductive window of age or are prepubertal (1,2). More than 90% of these cancer patients undergo invasive procedures of cancer therapy such as chemo and radiotherapy (3). 

Proliferative and active organs such as gonads are very sensitive to chemotherapy drugs and radioactive radiations. Infertility results from these treatments in most cases, especially when the treatment is in the abdominal and pelvic regions (4,5). More than 80% of prepubertal girls subjected to cancer treatments experience gonadotoxicity, which results in premature ovarian failure (POF) (6) and also the side effects of cancer treatment have recently gained a worldwide ubiquitous interest among different bio-medical scientific researchers (7). 

However, fertility preservation is not a matter of simple judgment (8). Nevertheless advances in cancer care and immediate monitoring of both physicians and patient in choosing a method of fertility preservation (9,10). 

### Cryostorage of gametes and gonadal tissues

Oocyte, embryo and ovarian tissue cryopreservation are being increasingly proposed for fertility preservation among cancer patients undergoing therapy, to enable them to have babies after the cancer cure (11). Until now, embryo cryostorage is the only established method in clinical practice. Embryo cryopreservation is not possible in a few cases, as in single girls who do not have or wish for a sperm partner yet and in prepubescent girls who do not yet possess a mature hypothalamus-pituitary-ovarian axis (12). Furthermore, oocyte retrieval is an extended procedure, which may not be possible in cases where cancer treatment cannot be delayed. Research on oocyte cryopreservation has shown promising results in animal models but it has not been accepted yet as a reliable procedure to save human female gametes (12,14). 

Preservation of ovarian tissues is a promising alternative to oocyte preservation because the ovarian tissue can be extracted using a simple laparoscopic procedure at any time, irrespective of menstrual cycle stage and age. It is thus better suited than oocyte preservation, for the specific cases mentioned above, for fertility preservation (15). 

Primordial follicles are the smallest female fertility unit, including 90% of the ovarian follicular reservoir (16). Although there are other types of follicles present in ovaries removed from the patient, primordial follicles are the ones that are considered for ovarian cryopreservation (17,18). Dormant primordial follicles are the most resistant of all follicles to cryo-injury because of the small size of their oocytes, small amounts of cytoplasmic lipids present, and absence of meiotic spindle within cytoplasm (19). These follicles are located anatomically in the ovarian cortex near the surface epithelium. To obtain best results for cryopreservation, it is best to remove the ovarian cortex from the medulla, which helps extreme penetration of cryoprotectants into the cortical tissue (20). Nevertheless some researchers have reported whole ovarian cryopreservation in animals (21) and humans (22). 

Different cryopreservation methods including cryostorage have been performed on biological samples. Among these, slow freezing has been the main procedure for preserving the ovarian tissue in liquid nitrogen (23,24) and recently vitrification has elicited interest as a reliable method for embryo cryopreservation in many fertility treatment centers (25). 

**Fig.1:**
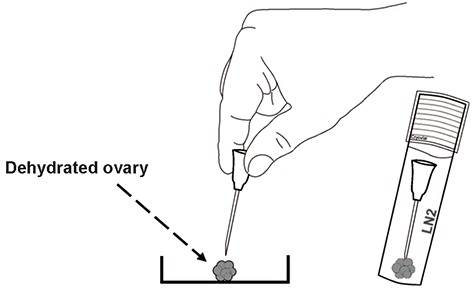
Using of cryopin in picking up of dehydrated ovary and plunging into the liquid nitrogen (18).

**Fig.2:**
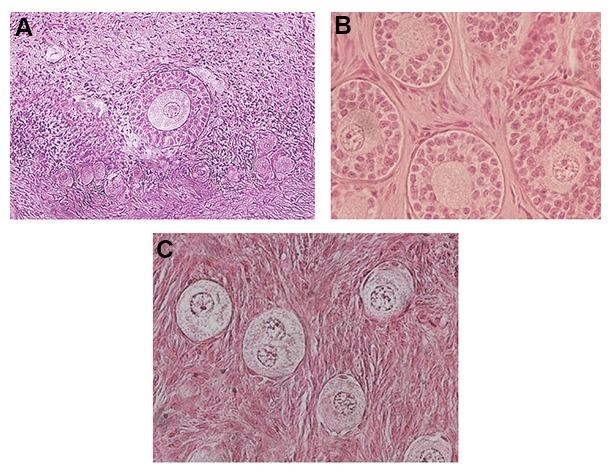
Vitrified ovarian tissues using of cryopin: A. Sheep, B. Monkey, and C. Human (Royan Ovarian Tissue Bank, Tehran, Iran).

To vitrify ovarian strips, Amorim et al. (26) applied the cryopin (freezing needle) procedure described by Fathi et al. (20), because promising results have already been obtained during vitrification of sheep ovarian tissue (Fig .1). Similar ovarian vitrification methods (needle immersion vitrification) (27) have also been reported in models of mouse (28) and human (28,29). Amorim and coworkers reported that ovarian fragments could be easily handled during dehydration, vitrification and reserving the tissue in cryovials using of cryopin (Fig .2) (26). 

### Gonad transplantation/re-implantation

**Fig.3:**
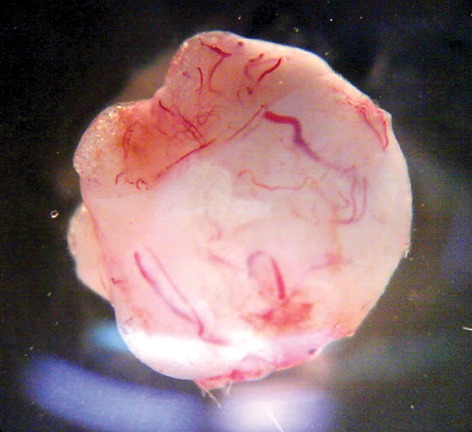
Re-angiogenesis 3 weeks after rat whole ovarian autotransplantation (Oocyte biology lab, Royan Institute).

The final goal of ovarian cryopreservation is re- implantation of the ovarian tissue in the patients (21,30) in order to re-establish the folliculogenesis cycle and activity of the reproductive hormones (Fig .3). Although this procedure dates back to the early years of the 18^th^ century (31), only recently have Donnez et al. (32) reported the first successful human live birth after transplantation of cryopreserved ovarian tissue. 

**Fig.4:**
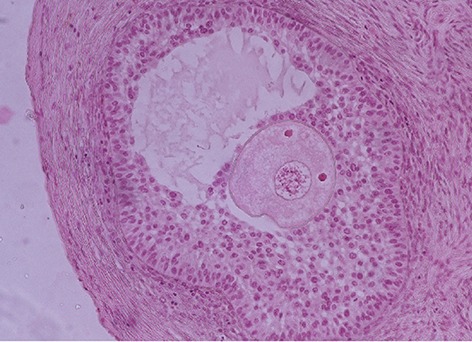
Developing a healthy antral follicle within Xenotransplanted sheep ovarian tissue into the male rat (33).

Although successful ovarian tissue re- implantation could help the patients who want to have children after cancer treatment, the recurren’ce of malignancy in its original location continues to be a concern (27). Cellular and molecular signaling from the recipient body are also major concerns in this procedure. Therefore there is much research being carried out to find strategies to induce follicular growth within the ovarian biopsies without grafting on to a recipient. Furthermore, delayed re-anastomosis affects the outcome of autotransplantation. Xenotransplantation or transplantation of the tissue into another species could possibly solve this problem. Hajimusa et al. (33) reported follicular development to antral stage, 8 weeks after sheep ovarian cortex xenotransplantation in rat (Fig .4). This result indicates the probability of successful xenograft of human ovarian tissue in future. Abtahi and her colleagues used therapeutic ultrasound to induce migration of endothelial cells toward the graft (34). They presented promising results in re-angiogenesis within ultrasound waves exposed transplanted mouse ovaries. 

### In vitro culture: simulation of in vivo

*In vitro* culturing is a promising route to obtain mature oocytes from cryopreserved ovaries or isolated follicles (35). Recently, Isachenko (1) compared the culture *in vitro* and the culture on embryonic chorioallantoic membrane (CAM) of cryopreserved human ovarian medulla-contained and medulla-free cortex. Theoretically, ovarian tissue culture is a safe and feasible method to produce mature fertilizable oocyte. A short- term culture of thawed ovarian pieces before xenografting can increase the number of growing follicles in the tissue (36). Difficulties in ovarian culture has hitherto made this a problematic procedure (37). Long folliculogenesis is the most important issue in large species, especially in humans. For example, in humans, an activated primordial follicle needs more than 300 days of growth and development to reach the mature preantral stage (38). 

Induction, long and powerful supports are essential to obtain mature follicles from ovarian cultured tissues (39). It must be noted that the *in vitro* developmental period is less than *in vivo *(40) but oocytes obtained from *in vitro* culture systems are often not fertile due to eliminated inhibitory factors in culture medium, which affects the quality of oocytes (41). The second challenge in this procedure is contamination of the medium during long term culture. 

Fatehi et al. (35) evaluated the effects of different ovarian vitrification protocols on the 2D culture of 12-day old mice preantral follicles. In this work, before plunging in liquid nitrogen, ovaries were first loaded into an acupuncture needle (NIV) or placed on a cold steel surface [solid surface vitrification (SSV)] for about 10 to 20 seconds. As a result, morphology and integrity of ovarian tissue were well-maintained, regardless of the vitrification protocol and NIV showed better follicular preservation after 12 day of culture than the SSV method. Gene expression patterns during culture could not explain the reduced survival rate observed in the solid surface group. 

### 3D culture systems: developing artificial organs

Despite using different types of antibiotics in the culture medium, the problem of the medium infection has remained unsolved. One other problem is the density and compaction of the cells and presence/growth of interstitial components in ovarian cortex (42), which do not allow nutrients to penetrate easily into this dense structure, resulting in damage to the tissue especially during the early stages of culture (43). Researchers have tried to optimize the tissue culture procedure and medium composition to overcome the above drawbacks. 

In the last decade, tissue engineering research has led to new culture methods to produce 3-dimensional artificial tissues. Reconstruction by using tissue background similar to physiologic conditions has been shown to result in better follicular development (44,45). Different types of biological scaffolds such as matrigel, alginate and fibrin materials are commonly used for cell (46), follicle (47) and tissue (48) 3D culture and transplantation. Some scientists have also reported 2 dimensional (2D) follicular culture without scaffolds (16,35). 

In 3D culture, the most important feature is to use a biological material that allows oxygen diffusion, distribution of nutrients and removal of cellular wastes. There is a critical need for oxygen delivery into the ovary where secondary and preantral follicles grow, especially when long-term *in vitro* cultures are needed (49). Mechanical features and properties of biological materials (biomaterials) such as density and concentration, stiffness, molecular weight and shaping (modeling) capacity are of particular importance in such procedures (50). 

Desai et al. (51) used a Tyramine-based hyaluronan (HA) hydrogel to culture fresh and vitrified preantral follicles from ovaries of 10- 12 days mice and demonstrated the potential role of this hydrogel in mitosis and generation of metaphase II (MII) oocyte. Amorim et al. (52) investigated the survival of human preantral follicles on calcium alginate matrix *in vitro* culture after isolation of follicles from frozen ovarian tissue. All thawed follicles showed an increase in size and 90% survival rate after 7 days culturing in alginate hydrogel system. 

Kedem et al. (53), compared alginate scaffolds with matrigel in culturing human primordial follicles of ovarian tissue. They showed that the number of developing follicles was significantly higher in samples cultured in alginate scaffolds in comparison with samples in Matrigel in which the number of atretic follicles was significantly higher than in samples that were cultured in the alginate scaffolds. Estradiol (E^2^) production was similar in both alginate and matrigel-cultured samples. 

Recently, Luyckx et al. (54) designed nine combinations of fibrinogen and thrombin as an artificial (synthetic) matrix for culturing human ovarian stromal cells, which maximize the dynamic density and minimize apoptosis of cells *in vitro*. Vitrification and ovarian tissue (organ) culture are approaches in which the population of primordial and primary developing follicles can be preserved besides conserving their complex communications (55). They enhance viability of cryopreserved tissues in extracellular matrix culture system (44) and preserve their morphological integrity leading to good survival and follicular growth and proliferation (56). 

Jin et al. (57) purposed a novel two-stage protocol to support growth of early primordial follicles and their capacity to produce mature oocytes for fertilization. They cultured ovaries of 8-day-old mice for 4 days and enhanced the population of primordial to primary and primary to secondary follicles to levels similar to those of a 12-day-old healthy mouse ovary. In the next step, they isolated secondary follicles and cultured them for 12 days in alginate alone or in alginate and fibrin combined matrix. Larger number of oocytes cultured in alginate fibrin matrix than pure alginate matrix progressed to metaphase-I and reached to metaphase-II could be fertilized and some could even cleave to 2-cell embryos. 

### Helping to egg maturation: adding supplements

Magalhães-Padilha et al. (58) investigated long-term culturing of goat ovarian tissue that was supplemented with different amounts of follicle stimulating hormone (FSH) and growth hormone (GH) in two stages of 0-8 day and 8-16 day periods. Among the 10 different treatments (α-MEM/α-MEM, FSH/FSH, FSH/GH, FSH/FSH+GH, GH/GH, GH/FSH, GH/FSH+GH, FSH+GH/FSH+GH, FSH+GH/FSH and FSH+GH/ GH), FSH/GH at day 16 of culture showed highest percentage of normal follicles, follicular activation, secondary follicles formation and also produced larger follicular diameter. 

Brito et al. (59) cultured ovarian cortical strips and evaluated viability of follicles and their molecular features. They compared culture conditions supplemented with beta mercaptoethanol (BME), BMP-4 or PMSG in short-term culture of 24 hours. 

Primordial follicles did not reach the primary stage with *in vitro* culture (IVC), but secondary follicle formation increased in culture systems containing the mentioned materials up to 44.86% compared to the control group, with the rate of 9.20%. 

King et al. (60) investigated surface epithelium regeneration in 3D cultured ovaries fragments (organoids) and fallopian tubes in alginate scaffolds. Tissues in control serum-free a-MEM medium formed only a single layer of cellular proliferation after 7 days of culturing, while supplementing medium with insulin induced hyper-proliferation and resulted in formation of several cell layers. Hilliard et al. (61) also showed the effects of gonadotropins on induction of ovarian surface epithelium (OSE) proliferation in 3D ovarian organ culture in alginate and 2D normal mouse cell lines in an 8-day culture system. They also demonstrated that use of Akt and epithelial growth factor (EGFR) inhibitors could block gonadotropin-induced proliferation. In conclusion, FSH and LH and a combination of these hormones increase cellular proliferation through activation (or induction) of Akt signaling and upregulating proliferative cyclin dependent kinases and anti- apoptotic Birc5. 

Parte and colleagues collected ovaries from a postmortem case of a 13 year old girl and one adult ovary from a peri-menopausal woman undergoing total abdominal hysterectomy. On culturing the cortical fragments of the ovaries in medium supplemented with FSH and basic fibroblast growth factor (bFGF) on Millicell-CM inserts for 3 days, they could induce a prominent proliferation of ovarian surface epithelium and transition of primordial follicles to primary (62). 

Wiedemann et al. (63) dissected ovaries from domestic cats and performed slow freezing protocol on 2 mm diameter pieces of ovarian cortex followed by a 14-day culture before and after cryopreservation. The integrity of primordial follicles was assessed by histological studies. During the culture, the number of primordial follicles decreased within the ovarian pieces and this effect was less observed when fetal bovine serum (FCS) was used instead of bovine serum albumin (BSA). Vitamin C supplementation had defective effect on follicles survival. Their cryopreservation protocol showed no deterioration of follicle survival after 1 week of culture. They could preserve a large quantity of follicles within the ovarian tissue using this slow freezing protocol in a variety of feline species. 

In 2013, Ki et al. (64), demonstrated that signals from insulin growth factor (IGF) and IGF-1 resulted in proliferation and hyperplasia of ovarian surface epithelium and did not decrease (affect) follicular integrity in response to up-regulation of PI 3-kinase pathway. Ovaries from CD1 mice were cultured in alginate hydrogels in the presence and absence of 5 μg/ml insulin or IGF-I for 7 days. Morphology of OSE was investigated by hematoxylin and eosin (H&E) and immunohistochemistry for cytokeratin 8 (CK8). BrdU was added to the medium 24 hours before fixation to assess proliferation. Culturing organoids in basic medium formed single squamous layer of OSE and showed little proliferation but supplementing (inclusion of) culture medium with IGF or IGF-I resulted in approximately 4-6 cell layers of hyperplastic OSE. Primordial follicles were observed in these cell layers. 

Many studies are now focusing on the use of growth factors to support cells and tissues during long-term *in vitro* culturing. These factors include bFGF (65), Kit-ligand (66), neurotrophins (67), IGF-I (68), IGF-II (69,70) and members of transforming growth factor-β (TGF-β) such as growth differentiation factor-9 (GDF-9), bone morphogenetic protein-15 (BMP-15) (71) and anti mullerian hormone (AMH) (72). Leukemia inhibitory factor (LIF) is a member of TGFβ superfamily that is expressed by pre-granulosa cells and promotes transition of primordial follicles to primary follicles, initiates oocyte development, proliferation and differentiation of theca cells from stromal tissue (73). BMP-6 is expressed in follicles and granulosa cells and also induces proliferation of granulosa cells and survival of follicles (74,75). 

Platelet derived growth factor (PDGF) is expressed (produced) by oocytes and along with neuregulin (NRG), vascular endothelial growth factor (VEGF) and EGF, acts as the extracellular factor in orchestrating transition of primordial to primary follicles (76,77). 

FSH as a gonadotropin was found to have positive effects on short (78) and long (79) term follicular and ovarian tissue *in vitro* culture. FSH is an endocrine factor that induces follicular growth and is necessary for production of steroids, differentiation of granulosa cells and formation of antrum (80). Although early follicular development is independent of gonadotropin, it has been shown that using of FSH and BFGF composition in ovarian cultures increased survival and development of the primordial follicles (81). It has recently been understood that FSH is the main proliferative and survival factor of granulosa cells. Attrition of isolated preantral follicles decreased in medium supplemented with FSH (82) and effectiveness of this hormone on *in vitro* (83) and *in vivo* (84) induction and survival of secondary follicles have been proved. GDF-9 and bFGF enhance the effect of FSH on survival, activity and growth of bovine primordial follicles (85). They could also increase percentage of primary follicles in all stages of 14- day *in vitro* culture and secondary follicles after 14-days of culturing. 

### Reactive oxygen species: one of the main problems

The changes in oxygen levels *in vitro* are more rapid and more than *in vivo* conditions that could lead to toxicity of the culture medium (86). It causes activation of reactive oxygen species (ROS) in it (87). Various antioxidants such as N-acetyl-L-cysteine (NAC) have been used to prevent the harmful effects of ROS on follicular development (83,88,89). Also acetylcysteine called N-acetylcysteine or NAC is a pharmaceutical drug and nutritional supplement used primarily as a mucolytic agent and in the management of paracetamol (acetaminophen) overdose (90). It is also used in clinical treatments such as controlling inflammatory responses, insulin resistance in diabetic patients (91,92) and in chronic lung diseases (93). In a study in which 25 mM concentration of NAC was used (88), healthy early preantral follicle was seen in cultured human ovarian pieces after 32 weeks. NAC also acts as a reductant and increases production of glutathione, the most abundant cellular thiol that removes intracellular peroxidase (94,95). However, the process of cell death can be regulated to some extent *in vitro*; some researchers were able to suppress ovarian tissue apoptosis using NAC (83). 

Mahmoodi et al. (89) investigated the effects of N-acetyl-L-cysteine as an antioxidant on mouse ovary heterotopic autotransplantation and reported considerable improvements in follicular survival and development and also in the structure and function of transplanted ovaries, through reduction of oxidative stress and apoptosis. They also studied the effect of erythropoietin (EPO) as an antioxidant on oxidative stress and ovary survival following transplantation. EPO increased follicle survival and function in grafted ovaries due to reduction of ischemia/reperfusion (IR) injury (96). 

Sadeu et al. (97) demonstrated the protective effect of NAC on follicular growth arrest induced by methoxychlor in 2006 and some years later in 2011. Maniu et al. (98) showed the protective effect of NAC against gentamicin toxicity on the 1-4 day-old rats cochlear culture. Its protective effect on boar sperm was also demonstrated. It was observed that the ROS level after freezing and thawing of sperms increased. Supplementing sperms with NAC (1 mM for 60 minutes) could increase capacitation and induction of acrosome reaction induced by addition of calcium ionophore a23187. Its application during sperm thawing and preparation for IVF could reduce DNA fragmentation and lipids peroxidation of the sperm (99). 

The protective potential of NAC was also tested on osteoblasts isolated from rat bone marrow by inducing oxidative stress (100 M H_2_O_2_) and treating with 2.5-5 mM of NAC. Cultures without NAC showed 50% reduction of cell number after 2 days. Addition of NAC could recover the expression of type I collagen, osteopontin and osteocalcin, which were down-regulated by H_2_O_2_ on day 7 of culture (100). 

Diethylhexyl phthalate (DEHP) has been shown to inhibit growth of mouse antral follicles. Ovaries of 31-35-day-old mice were cultured with DMSO or Di (2-ethylhexyl) phthalate (DEHP) with or without NAC. Results showed that NAC (1-10 mM) could block the ability of DEHP to inhibit follicular growth, increase ROS and reduce expression and activity of Cu/Zn superoxide dismutase antioxidant enzymes (101). Because of these promising results, we at the Royan Institute (Iran), have focused on short- and long- time culturing of ovary and have observed several noticeable effects of NAC during this procedure in mouse (unpublished data). 

## Conclusion

With the worldwide increase in the number of young female cancer patients receiving invasive therapy, there has been increased focus on development of fertility preservation techniques. 

Autotransplantation of preserved gonadal tissues is a solution, but recurrence of malignancy to the transplanted ovarian tissues continues to be a serious concern. While xenografting is a promising alternative, molecular signal transduction from recipients that can cause complications. *In vitro* culturing and making artificial gonads can overcome the above problems and this paper has reviewed pertinent research in this area. 
